# Obstructive Sleep Apnea and Coronary Artery Disease: An Overlooked Cardiovascular Risk Factor

**DOI:** 10.3390/biomedicines14030515

**Published:** 2026-02-26

**Authors:** Nardi Tetaj, Andrea Segreti, Michele Pelullo, Virginia Ligorio, Martina Ciancio, Aurora Ferro, Riccardo Cricco, Simone Pasquale Crispino, Gian Paolo Ussia, Francesco Grigioni

**Affiliations:** 1 Cardiology Unit, Fondazione Policlinico Universitario Campus Bio-Medico, Via Alvaro del Portillo 200, 00128 Roma, Italy; nardi.tetaj@unicampus.it (N.T.); michele.pelullo@unicampus.it (M.P.); virginia.ligorio@unicampus.it (V.L.); martina.ciancio@unicampus.it (M.C.); aurora.ferro@unicampus.it (A.F.); riccardo.cricco@unicampus.it (R.C.); simone.crispino@unicampus.it (S.P.C.); g.ussia@policlinicocampus.it (G.P.U.); f.grigioni@policlinicocampus.it (F.G.); 2 Research Unit of Cardiovascular Science, Department of Medicine and Surgery, Università Campus Bio-Medico di Roma, Via Alvaro del Portillo 21, 00128 Roma, Italy

**Keywords:** obstructive sleep apnea (OSA), coronary artery disease (CAD), sleep-disordered breathing, atherosclerosis, myocardial infarction, continuous positive airway pressure (CPAP), cardiovascular risk factors, hypoxic burden

## Abstract

Obstructive sleep apnea syndrome (OSA) is increasingly recognized as a common and clinically relevant comorbidity in coronary artery disease (CAD). Epidemiological studies demonstrate that OSA is highly prevalent among patients with CAD and independently increases the risk of myocardial infarction, accelerated atherosclerosis, and recurrent adverse events. The pathophysiological mechanisms underlying this association include intermittent hypoxia, sympathetic overactivation, oxidative stress, endothelial dysfunction, systemic inflammation, metabolic dysregulation, and pro-prothrombotic changes. These processes converge to promote coronary plaque formation, instability, and ischemia. Clinical evidence indicates that OSA contributes to silent nocturnal ischemia, higher rates of acute coronary syndromes, restenosis after percutaneous coronary intervention, and worse prognosis following myocardial infarction or surgical revascularization. Continuous positive airway pressure (CPAP) therapy improves blood pressure, endothelial function, and surrogate markers of ischemia, but large randomized trials have yielded neutral results on major cardiovascular events, largely due to suboptimal adherence. However, observational studies, however, suggest improved survival in patients who are adherent to CPAP therapy. Lifestyle interventions, particularly weight reduction, remain essential adjunctive strategies. This review synthesizes current evidence, evaluates therapeutic implications, and highlights the need for systematic OSA screening in CAD populations. Future research should focus on patient phenotyping, treatment adherence, and integrated care models to improve cardiovascular outcomes.

## 1. Introduction

Obstructive sleep apnea (OSA) is a prevalent sleep-related breathing disorder characterized by recurrent episodes of partial or complete upper airway obstruction during sleep, resulting in intermittent hypoxemia, hypercapnia, arousals, and marked sympathetic activation. OSA arises from the combined influence of upper airway anatomy and impaired neuromuscular control of pharyngeal patency during sleep [1]. Population studies estimate that moderate to severe OSA affects approximately 10–20% of middle-aged adults, with prevalence rising substantially in individuals with obesity, hypertension, and cardiovascular comorbidities [2]. Despite this high burden, OSA remains underdiagnosed, particularly among patients with cardiovascular disease.

OSA is diagnosed on the basis of symptoms (snoring, arousals, breathing pauses, daytime sleepiness and fatigue) and overnight polysomnography or home sleep apnea testing (HSAT) study, which measure apnea–hypopnea index (AHI) and respiratory event index (REI), respectively. The AHI represents the number of obstructive respiratory events per hour of sleep, such as apnea (≥10 s) and hypopnea (reduction in breathing with a reduction in SpO_2_ ≥ 4%). The REI represents the number of obstructive respiratory events per hour of estimated sleep. Diagnosis requires an AHI or REI ≥ 5 in the presence of symptoms, or ≥15 regardless of symptoms. The AHI categorizes the severity of OSA, from mild (AHI 5–14.9) to moderate (AHI 15–29.9) and severe form (AHI > 30) [3,4].

Coronary artery disease (CAD) continues to represent the leading cause of morbidity and mortality worldwide. Traditional risk factors such as dyslipidemia, hypertension, diabetes mellitus, obesity, and smoking are well established [5]. However, emerging evidence underscores the importance of OSA as an independent and potentially modifiable contributor to coronary risk. Epidemiological studies and meta-analyses consistently demonstrate that OSA is associated with a higher incidence of myocardial infarction, accelerated atherosclerosis, and increased long-term mortality [6]. Moreover, the severity of OSA, quantified by the apnea–hypopnea index (AHI) or nocturnal oxygen desaturation burden, shows a dose–response relationship with coronary outcomes [7].

The biological plausibility of this association is supported by multiple mechanistic pathways. Intermittent hypoxia and sleep fragmentation trigger sympathetic overactivity, oxidative stress, endothelial dysfunction, systemic inflammation, and prothrombotic changes, all of which are recognized contributors to atherogenesis and coronary plaque instability [8]. Clinically, OSA not only predisposes the development of coronary artery disease but also adversely affects prognosis after acute coronary syndromes and revascularization procedures [9,10].

In this review, we provide a comprehensive synthesis of current evidence linking OSA and CAD. We examine epidemiological associations, dissect key pathophysiological mechanisms, evaluate clinical outcomes, and discuss the impact of OSA treatment on coronary risk. Finally, we highlight ongoing controversies, unmet needs, and future research directions in this evolving field.

### Search Strategy and Selection Criteria

A comprehensive literature search was performed on PubMed covering the period from 2000 to September 2025. The search strategy used the terms obstructive sleep apnea, sleep disorder, and sleep apnea in combination with key terms related to coronary artery disease, cardiovascular diseases, atherosclerosis, inflammation, myocardial infarction, myocardial ischemia, arrhythmias, dyslipidemia, diabetes, thrombosis, heart failure, hypertension, and the management of OSA (CPAP and BiPAP). We excluded proceeding papers, corrections, early access articles, news items, book chapters, retractions, reprints, biographical items, book reviews, meeting abstracts, editorial materials, and letters. We included relevant English-language studies involving adult populations, with particular focus on randomized controlled trials and large observational studies. The full texts were then screened and selected, and the study characteristics and information were extracted from the selected studies. A flowchart illustrating the selection process is provided in the Supplementary Material, Figure S1.

## 2. Epidemiology of OSA

The epidemiological link between obstructive sleep apnea (OSA) and coronary artery disease (CAD) has been consistently demonstrated across observational studies, registries, and meta-analyses. In population-based cohorts, the prevalence of moderate to severe OSA (apnea–hypopnea index, AHI ≥ 15 events/h) is estimated at 10% to 20% in middle-aged adults, with higher rates among men, older individuals, and those with obesity [11,12]. Conversely, in patients with CAD, the prevalence is considerably greater. Studies of patients undergoing coronary angiography or revascularization procedures report that 30–60% have unrecognized OSA, underscoring the overlap between these conditions [13]. Importantly, the greatest health burden of OSA lies in its strong association with cardiovascular diseases, including systemic hypertension, coronary artery disease, heart failure, and stroke [14]. Longitudinal data confirm the growing scope of this problem. Using the Wisconsin Sleep Cohort, Peppard et al. documented a substantial rise in sleep-disordered breathing (SDB) prevalence in U.S. adults aged 30–70 years between 1988–1994 and 2007–2010 [15]. The prevalence of moderate-to-severe SDB (AHI ≥ 15) increased to 10% in men aged 30–49, 17% in men aged 50–70, 3% in women aged 30–49, and 9% in women aged 50–70. Overall, prevalence rose by 14–55% across subgroups, largely paralleling the obesity epidemic, highlighting SDB as a growing public health burden with major implications for cardiovascular and metabolic risk [15].

## 3. Pathophysiological Mechanisms

The association between obstructive sleep apnea (OSA) and coronary artery disease (CAD) is biologically plausible and underpinned by multiple, interrelated pathophysiological processes. Recurrent episodes of upper airway obstruction during sleep lead to cycles of intermittent hypoxemia, sympathetic overactivation caused by arousals and sleep interruptions, and intermittent increase in negative intrathoracic pressure caused by the obstructive of the upper airway tract, leading to hypercapnia and a cascade of downstream effects that promote atherosclerosis, thrombosis, and myocardial ischemia [16,17].

### 3.1. Endothelial Dysfunction and Inflammation

A hallmark of OSA is intermittent hypoxia, which resembles repeated ischemia–reperfusion injury at the vascular level, contributing to oxidative load through several pathways, each reinforcing the other in a vicious cycle that sustains vascular injury.

These pathways are not independent: they form a feed-forward network. The net result is sustained oxidative stress, vascular inflammation, and a pro-atherogenic environment that contributes to plaque formation and atherosclerosis [18].

**Figure 1:**
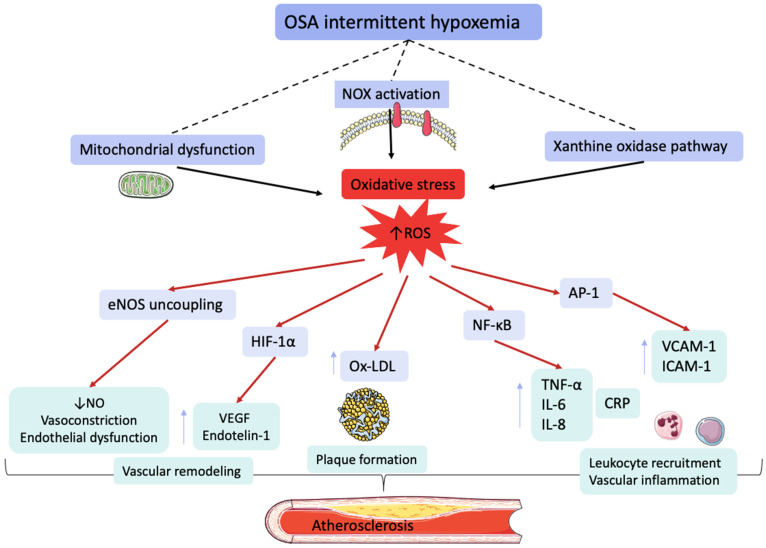
Pathways involved in OSA endothelial damage due to oxidative stress. Abbreviations: OSA, obstructive sleep apnea; NOX, NADPH oxidases; eNOS, endothelial nitric oxide synthase; ROS, reactive oxygen species; HIF-1, Hypoxia Induced Factor-1; ox-LDL, oxidized low-density lipoproteins; NO; VEGF; NF-κB, nuclear factor-κB; TNF-α, tumor necrosis alfa; IL-6 and IL-8, interleukin 6 and 8; CRP, C-reactive protein; VCAM-1, vascular adhesion protein 1; ICAM-1, intracellular adhesion molecule 1; AP-1, activator protein 1; blue up-ward arrow, increase; the red arrows, effect caused by the increase of ROS.

Reoxygenation after each apneic event causes transient mitochondrial hyperpolarization leading to accumulation of reactive oxygen species (ROS) such as superoxide anion (O_2_^−^) and hydrogen peroxide (H_2_O_2_), which can diffuse across membranes and propagate oxidative stress. Persistent chronic intermittent hypoxia alters mitochondrial dynamics (fusion/fission balance), impairs oxidative phosphorylation efficiency, and damages mitochondrial DNA, perpetuating mitochondrial dysfunction and ROS overproduction [10], Figure 1.

NADPH oxidases (NOX) catalyze the transfer of electrons from NADPH to oxygen, producing superoxide. Chronic intermittent hypoxia upregulates NOX2 and NOX4 in vascular endothelial cells, smooth muscle cells, and circulating leukocytes. This process is enhanced by sympathetic activation and angiotensin II signaling, both elevated in OSA, amplifying redox imbalance [19].

The xanthine oxidoreductase system (which includes xanthine dehydrogenase and xanthine oxidase) contributes significantly to ROS during hypoxia–reoxygenation. Under hypoxic conditions, xanthine dehydrogenase is converted to xanthine oxidase, which, upon reoxygenation, metabolizes hypoxanthine in uric acid, generating superoxide and hydrogen peroxide as byproducts. Elevated serum uric acid levels, frequently observed in OSA patients, have shown to be indirect markers of xanthine oxidase activation and oxidative stress burden [20].

Under physiological conditions, endothelial nitric oxide synthase (eNOS) produces nitric oxide (NO), a vasoprotective molecule that promotes vasodilation and inhibits platelet aggregation. Oxidative environments lead to endothelial nitric oxide synthase (eNOS) uncoupling. Instead of producing NO, uncoupled eNOS generate superoxide, reducing the production of NO and leading to vasoconstriction and inflammation, key processes in early atherogenesis, exacerbating endothelial dysfunction and amplifies oxidative stress [18].

ROS attack unsaturated fatty acids in cellular and plasma membranes, leading to lipid peroxidation, and generation of reactive aldehydes such as malondialdehyde (MDA) and 4-hydroxynonenal (4-HNE). These products modify low-density lipoproteins (LDL) into oxidized LDL (oxLDL), which are avidly taken up by macrophages through scavenger receptors, forming foam cells, the cornerstone of atherosclerotic plaque formation [21].

ROS activate nuclear factor-κB (NF-κB), enabling nuclear translocation and transcription of IL-6, TNF-α, IL-8, and CRP [22]. This enhances systemic inflammation and endothelial activation [23,24].

Intermittent hypoxia increases the production of Hypoxia Induced Factor-1α (HIF-1α), leading to increased transcription of Vascular endothelial growth factor (VEGF), endothelin-1, and erythropoietin, contributing to vascular remodeling and sympathetic drive [25].

Sies et al. showed that HIF-α is activated by a different pathway in intermittent hypoxia than in prolonged hypoxia [19].

ROS stimulate activator protein 1 (AP-1) to induce matrix metalloproteinases and adhesion molecules such as intracellular adhesion molecule 1 (ICAM-1), vascular cell adhesion protein 1 (VCAM-1), while paradoxically suppressing the Nrf2-antioxidant response element (ARE) system, which normally upregulates superoxide dismutase (SOD), catalase, and glutathione peroxidase. Together, these transcriptional changes drive endothelial activation, leukocyte recruitment, and vascular inflammation, amplifying cardiovascular risk [26,27].

This oxidative stress promotes vascular inflammation mediated by leukocyte activation and adhesion to the endothelium, playing a central role in the formation of atherosclerotic plaques. Activation of monocytes and T lymphocytes leads to the release of inflammatory mediators such as tumor necrosis factor α (TNF-α), interleukins 6 and 8 (IL-6, IL-8) and adhesion molecules such as intracellular adhesion molecule 1 (ICAM-1), vascular cell adhesion protein 1 (VCAM-1), L-selectins and E-selectin [28,29,30,31].

### 3.2. Prothrombotic State

In parallel, OSA is associated with increased fibrinogen and plasminogen activator inhibitor-1 levels, impaired fibrinolysis, enhanced platelet activity, and increased blood viscosity—all of which heighten the risk of intracoronary thrombosis and acute coronary events, particularly in the presence of vulnerable plaques [32,33].

### 3.3. Metabolic Dysfunction

Metabolic dysfunction provides an additional mechanistic link. Observational studies demonstrate strong associations between OSA and insulin resistance, even after adjusting for shared risk factors [24]. Intermittent hypoxia and inflammation interfere with insulin signaling, worsening glucose intolerance and promoting endothelial dysfunction and prothrombotic activity [34,35].

Insulin resistance also drives adipose tissue lipolysis, increasing free fatty acid release; combined with dysregulated lipid metabolism and impaired β-oxidation, this promotes hepatic triglyceride accumulation and steatosis, potentially progressing to fibrosis [36,37,38].

### 3.4. Sympathetic Overactivity

Sympathetic overactivity represents another hallmark of OSA. Recurrent arousals and hypoxemia induce sustained sympathetic activation, causing tachycardia, elevated vascular resistance, and increased myocardial oxygen demand. These hemodynamic perturbations accelerate endothelial injury and the progression of coronary artery disease [39].

Notably, this nocturnal overactivity extends into daytime hours, contributing to reduced heart-rate variability, persistent hypertension, arrhythmias, and heightened coronary shear stress. Reduced heart-rate variability itself is an established predictor of adverse cardiovascular outcomes, including arrythmias and sudden cardiac death [40,41,42].

OSA-related hypoxemia and hypercapnia also stimulate carotid body chemoreceptors, driving chemoreflex hypersensitivity and peripheral vasoconstriction. This results in greater dispersion of ventricular repolarization and upregulation of left ventricular calcium channels, enhancing arrhythmogenic potential [43,44,45]. Conversely, repetitive hypoxia and arousals impair baroreflex sensitivity, further predisposing patients to hypertension [46].

The cerebrovascular system is similarly affected. Recurrent arousals from sleep, triggered by increased respiratory effort to restore airway patency, cause sleep fragmentation and reduced sleep quality, contributing to excessive daytime sleepiness and cognitive impairment commonly observed in patients with OSA. In parallel, chronic intermittent hypoxia and recurrent surges in blood pressure promote endothelial dysfunction and accelerate atherosclerosis within the cerebral vasculature, thereby heightening the risk of stroke and other cerebrovascular events [47,48].

Concurrently, impaired cerebral autoregulation and persistent sympathetic overdrive exacerbate cognitive decline and favor the development of vascular dementia [49,50,51], while CPAP therapy has been shown to restore cerebral perfusion and improve cognition [52].

### 3.5. Increase Negative Intrathoracic Pressure

In healthy individuals, inspiratory intrathoracic pressure is typically around −8 cmH_2_O. In patients with OSA, apnea and hypopnea cycles generate repetitive Müller maneuvers (inspiratory efforts against an occluded airway), producing exaggerated negative intrathoracic pressures that can exceed −30 cmH_2_O. These hemodynamic shifts alter cardiac transmural pressures, leading to increased venous return to the right heart, elevated LV afterload, reduced LV compliance, and heightened myocardial wall stress. Over time, such mechanical stressors contribute to structural remodeling and may promote both atrial and ventricular arrhythmogenesis. Indeed, OSA has been established as both a substrate and trigger for atrial fibrillation [53,54].

**Figure 2:**
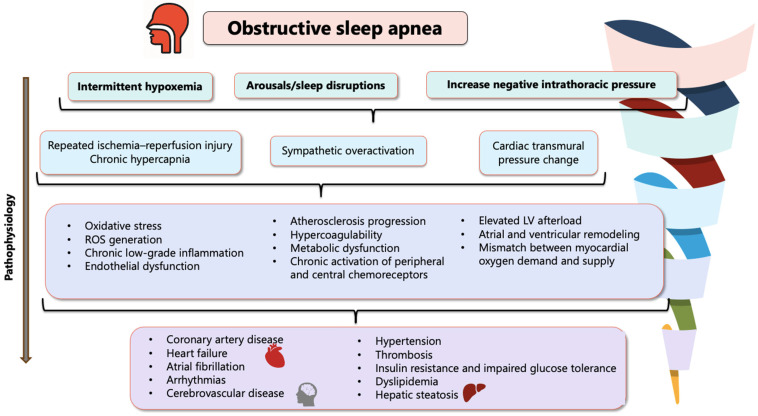
Pathophysiological mechanisms of obstructive sleep apnea in developing cardiovascular and secondary diseases. Abbreviations: ROS, reactive oxygen species; LV, left ventricular.

In addition, chronic sympathetic overactivity in patients with OSA further contributes to myocardial remodeling and dysfunction, Figure 2. The sustained sympathetic tone, combined with recurrent hypoxic episodes, increases cardiac workload and fosters the development of LV hypertrophy and diastolic dysfunction. Over time, these maladaptive structural and functional changes may progress toward overt heart failure [55,56,57].

Finally, OSA patients frequently experience silent myocardial ischemia during sleep, attributable to recurrent hypoxemic episodes, surges in sympathetic tone, and increased myocardial oxygen demand. ST-segment depression and ventricular ectopy occur most frequently during REM sleep, when apneic episodes are longest and most severe, frequently in the absence of angina. Such subclinical ischemic episodes contribute to the under-recognition of coronary involvement in OSA [58,59].

## 4. Clinical Evidence Linking OSA and Cardiovascular Diseases

The convergence of oxidative stress, hemodynamic strain, endothelial injury, inflammation, metabolic dysfunction, and hypercoagulability accelerates the development of coronary atherosclerosis and predisposes patients to acute ischemic events. Importantly, these mechanisms are synergistic and self-perpetuating, with intermittent hypoxia serving as the central driver that amplifies all pathological pathways.

Obstructive sleep apnea (OSA) represents a major, underrecognized health burden, underscoring the need for public health strategies to improve detection, case-finding, and treatment.

### 4.1. OSA and Coronary Artery Diseases

Several studies have consistently demonstrated an association between OSA and cardiovascular diseases, as shown in Table 1. OSA is highly prevalent yet frequently underdiagnosed, and has been linked to hypertension, coronary artery disease (CAD), stroke, metabolic dysfunction, and impaired quality of life [60].

Zhang et al., in a prospective, cross-sectional study of 137 patients hospitalized with unstable angina who underwent overnight polysomnography the night before invasive coronary angiography [61]. Patients with a high sleep apnea-specific hypoxic burden (SASHB) of ≥ 18% min/h had a significantly higher Gensini Score and SYNTAX Score than those with a low SASHB, in both univariate and multivariate adjusted model (adjusted for age, sex, smoking, hypertension, HbA1c, LDL). Notably, high SASHB also correlated with ≥50% right coronary artery stenosis [61].

Likewise, Barbé et al., performed an observational ancillary study of the ISAACC trial [62]. In 431 patients hospitalized for acute coronary syndrome who underwent sleep testing within 48–72 h, those with OSA (AHI > 15; *n* = 213) and no previous treatment with CPAP, showed greater ACS severity than controls (*n* = 218). Those patients with an AHI ≤ 15 events/h were considered controls. After adjustment (age, BMI, smoking, hypertension), OSA was linked to higher peak troponin; increasing AHI quartiles tracked with more diseased coronary vessels and higher troponin. Coronary Care Unit stay was longer with OSA, but total hospital days, in-hospital complications, and mortality were similar [62].

Furthermore, Shah et al. conducted a prospective, clinic-based cohort study of 1436 adults aged ≥50 years referred for suspected sleep-disordered breathing (1997–2001) who underwent attended polysomnography; OSA was defined as AHI ≥ 5 events/h (4% desaturation rule) [63]. Over a median 2.9 years of follow-up, OSA independently predicted the composite of myocardial infarction, coronary revascularization, or cardiovascular death (adjusted HR 2.06; 95% CI 1.10–3.86; *p* = 0.024), with a clear dose–response across severity strata versus AHI < 5 (AHI 5–14: HR 2.22; 15–29: 2.65; ≥30: 2.82; trend *p* = 0.005) [63].

Multiple observational studies have reported a significant association between obstructive sleep apnea (OSA) and coronary artery disease. Nevertheless, the majority have evaluated a composite outcome such as major cardiovascular events, including myocardial infarction, revascularization, and cardiac death [64].

**Table 1:** Key observational studies assessing the risk of major cardiovascular outcomes in patients with OSA compared with those without OSA. Abbreviations: OSA, obstructive sleep apnea; RDI, respiratory disturbance index; CAD, coronary artery disease; CVD, cardiovascular disease; HF, heart failure; CV, cardiovascular; PCI, percutaneous coronary intervention; AHI, apnea-hypopnea index; MACE, major adverse cardiovascular events; CI, confidence interval.

Study	Study Size	Population	OSA Severity	Follow-Up	Key Findings
Peker et al. (2000) [65]	62	CAD patients	RDI ≥ 10/h	5 years	CV mortality: 37.5% OSA vs. 9.3% non-OSA (*p* = 0.018)
Mooe et al. (2001) [66]	408	Patients with CAD undergoing angiography	AHI ≥ 10	5.1 years	Disordered breathing increased composite risk (death, stroke, MI). Strongest association with stroke.
Shahar et al. (2001) [67]	6424	Community adults who had in-home polysomnography of the Sleep Heart Health Study	AHI ≥ 11	Cross-sectional	AHI > 11 were associated with greater odds of prevalent CVD (myocardial infarction, angina, coronary revascularization procedure, heart failure, or stroke) after multivariable adjustment. Associations were stronger for heart failure and stroke than for coronary heart disease.
Marin et al. (2005) [68]	1651	Men with untreated OSA, healthy controls	AHI ≥ 30	10 years	Untreated severe OSA significantly increased the risk of fatal and non-fatal cardiovascular events compared with healthy participants. CPAP treatment reduced this risk.
Gami et al. (2007) [54]	3542	Adults with no prior AF	AHI ≥ 5	4.7 years	OSA independently predicted AF.
Gottlieb et al., (2010) [69]	4422	Adults ≥ 40 years and free of CVD/HF at baseline	AHI ≥ 30/h	8.7 years	After adjustment for multiple risk factors, OSA was a significant predictor of incident coronary heart disease (myocardial infarction, revascularization procedure, or coronary heart disease death) only in men age ≤ 70 years, but not in older men or in women of any age.
Shah et al. (2010) [63]	1436	1436 patients ≥ 50 years old referred for suspected SDB, mean follow-up 2.9 years	AHI ≥ 5	2.9 years	OSA independently increased risk of coronary events (MI, coronary revascularization, CV death).
Hla et al. (2015) [70]	1131	1131 adults without baseline CVD/HF, followed up to 24 years	AHI > 30	24 years	Severe SDB (AHI ≥ 30) associated with increased risk of CVD/HF (defined by new reports of myocardial infarction, coronary revascularization procedures, congestive heart failure, and cardiovascular deaths).
Barbé et al. (2015) [62]	431	431 patients with ACS (ancillary of the ISAACC trial)	AHI ≥ 15/h	Cross-sectional	Moderate-to-severe OSA were associated with ≥3 diseased vessels.
Lee et al. (2016) [71]	1311	Patients who completed a sleep study within 7 days of PCI.	AHI ≥ 15/h	1.9 years	OSA independently associated with higher MACCE risk (CV mortality, MI, stroke, revascularization), independently of age, sex, ethnicity, body mass index, diabetes mellitus, and hypertension.
Zhang et al. (2024) [61]	137	Patients with unstable angina undergoing angiography	AHI ≥ 15–21/h	Cross-sectional	High sleep apnea–specific hypoxic burden independently associated with severe CAD, assessed with Gensini and SYNTAX score.
Mazzotti et al. (2025) [7]	4396	Community adult patients free of CVD	AHI ≥ 5/h	11 years	Excessively sleepy OSA subtype had higher MACE risk (a composite of all-cause mortality, acute myocardial infarction, stroke and unplanned coronary revascularization), independent of hypoxic burden; hypoxic burden predicted CV mortality.

### 4.2. OSA and Other Cardiovascular Outcomes

In the Sleep Heart Health Study, Shahar et al. evaluated 6424 adults and found that moderate-to-severe sleep-disordered breathing (SDB) was associated with a higher prevalence of cardiovascular disease, particularly heart failure (OR 2.38) and stroke (OR 1.58). These findings established SDB as an independent cardiovascular risk factor in the general population [67].

In a prospective cohort of 408 patients ≤ 70 years with angiographically confirmed CAD, Mooe et al. reported that SDB (oxygen desaturation index ≥ 5 or AHI ≥ 10) was associated with a 60–70% increased risk of death, myocardial infarction (MI), or stroke over five years of follow-up. The strongest association was observed with stroke, where risk was nearly tripled, confirming SDB as an independent adverse prognostic factor in CAD [66].

The Wisconsin Sleep Cohort provided further evidence. Arzt et al. showed that 1475 individuals with moderate-to-severe SDB (AHI ≥ 20) had more than four-fold increased odds of stroke after four years, independent of confounders [72]. This study was an important prospective epidemiologic analysis suggesting that SDB may precede and contribute to stroke [72]. Similar results were found in the Sleep Heart Health Study in 2010 by Redline et al. [73]. In a subsequent 24-year follow-up, Hla et al. demonstrated that severe SDB (AHI ≥ 30) was associated with a two-fold higher risk of incident CAD or heart failure, further supporting SDB as a long-term cardiovascular risk factor [70].

In patients undergoing percutaneous coronary intervention (PCI), the multinational Sleep and Stent Study (Lee et al.) found that OSA (AHI ≥ 15) was present in 45% of 1311 patients and independently predicted major adverse cardiac and cerebrovascular events (MACCE), with a hazard ratio of 1.57 over 1.9 years of follow-up. The increased risk was driven mainly by higher cardiovascular and all-cause mortality, highlighting OSA as a frequent and adverse prognostic factor after PCI [71].

Similarly, Gottlieb et al., 2010, in a cohort 4422 adults without baseline CAD or heart failure from the Sleep Heart Health Study, observed that severe OSA was associated with increased risk of incident CAD and heart failure in men, but not in women [69]. More recently, in 2025, Mazzotti et al. showed that, over a median 12-year follow-up, the excessively sleepy OSA subtype predicted a higher risk of major adverse cardiovascular events (HR 1.6), while hypoxic burden independently predicted cardiovascular mortality [7].

Beyond coronary outcomes, OSA has been linked to atrial fibrillation (AF). In a population-based cohort of 3542 adults, Gami et al. demonstrated that OSA independently increased the risk of incident AF over 4.7 years [54]. More recent work, by López-Gálvez et al. in 2023, has further explored molecular mechanisms underlying postoperative AF in patients with OSA [74].

OSA has also been implicated in metabolic disease. Kendzerska et al., in a cohort of 8678 adults without diabetes, found that severe OSA (AHI > 30) was independently associated with a 30% higher risk of incident diabetes after 5.6 years. Nocturnal hypoxemia, reduced sleep duration, and higher resting heart rate further predicted diabetes risk [75]. Similarly, Lindberg et al., in a community cohort of 141 men followed for 11 years, showed that SDB predicted worsening insulin resistance and incident diabetes (OR 4.4 for ODI > 5) [76].

**Figure 3:**
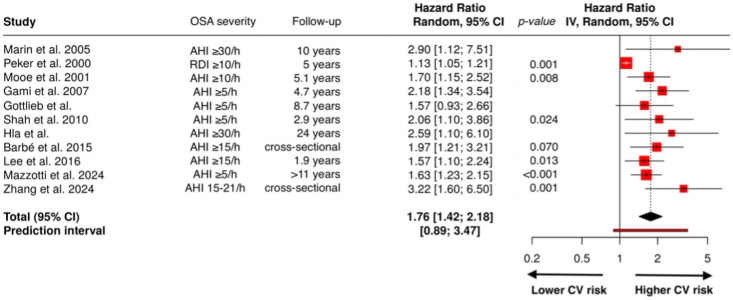
Forest plot showing the risk of major cardiovascular outcomes reported in key observational studies [54,61,62,63,65,66,68,69,70,71,77], comparing patients with OSA versus those without OSA. Abbreviations: OSA, obstructive sleep apnea; CV risk, cardiovascular risk; AHI, apnea-hypopnea index; RDI, respiratory disturbance index; CI, confidence interval.

Together, these findings provide robust population-level evidence that moderate-to-severe OSA is strongly linked to the development and progression of cardiovascular disease, supporting its recognition as a major and underdiagnosed cardiovascular risk factor, Figure 3.

## 5. Impact of CPAP Treatment

Given the strong association between obstructive sleep apnea (OSA) and coronary artery disease (CAD), an important clinical question is whether OSA treatment improves cardiovascular outcomes. Therapeutic strategies include continuous positive airway pressure (CPAP), weight loss, and lifestyle modification, with CPAP remaining the cornerstone therapy. CPAP effectively eliminates apneic events, improves oxygenation, and attenuates nocturnal sympathetic surges. Meta-analyses suggest that CPAP can lower inflammatory markers such as CRP, IL-6, and TNF-α, though effects vary across studies [78,79].

### 5.1. Observational Studies About CPAP Treatment

Large observational studies, both retrospective and prospective, show a benefit of CPAP in OSA patients in reducing the incidence of cardiovascular diseases during follow-up, highlighting OSA as a potentially modifiable cardiovascular risk factor. These findings support the notion that treatment adherence is essential and that real-world outcomes may diverge from trial results when compliance is higher [80], Table 2.

Marin et al., in a 10-year cohort study of 1651 middle-aged men, found that untreated severe OSA (AHI ≥ 30) was associated with a threefold higher risk of fatal and non-fatal cardiovascular events, including MI and stroke [68]. In contrast, CPAP-treated patients had outcomes similar to controls, highlighting the protective effect of therapy [68]. Mazzotti et al. in a Medicare cohort study of 888,835 adults with OSA (median follow-up 3.1 years) found that CPAP initiation was associated with lower all-cause mortality (HR 0.53) and lower MACE (HR 0.90), defined as a composite of first occurrence of myocardial infarction (MI), heart failure (HF), stroke, or coronary revascularization [77]. In the Swedevox registry of 16,425 OSA patients, non-adherence to CPAP (usage < 4 h/night) at 1 year predicted higher subsequent mortality over a median 2.4 years (adjusted HR 1.73, 95% CI 1.30–2.29) compared with adherent users (≥4 h/night) [81]. Similarly, Gervès-Pinquié et al., in a French cohort included 5138 adults with OSA starting PAP and followed a median 6.6 years [82]. Device-verified adherence (PAP ≥ 4 h/night) showed a dose–response association with lower risk of MACE (death, MI, stroke, HF exacerbation, or revascularization) versus non adherent (<4 h/night), driven mainly by lower all-cause mortality [82].

**Table 2:** Observational studies and randomized controlled trials assessing the risk of major cardiovascular outcomes in patients with OSA treated with CPAP versus those untreated with CPAP. Abbreviations: OSA, obstructive sleep apnea; CPAP, continuous positive airway pressure; ACS, acute coronary syndrome; CAD, coronary artery disease; HF, heart failure; MI, myocardial infarction; TIA, transient ischemic attack; UA, unstable angina; CV, cardiovascular; AHI, apnea-hypopnea index; ESS, Epworth Sleepiness Scale score; MACE, major adverse cardiovascular events.

Study	Design	Population	Follow-Up	Sample Size	Exposure/Intervention	Main Finding (CV Endpoints)
Palm et al., 2018 [81]	Observational study	Swedevox Swedish national registry of OSA patients on CPAP.	2.4 years	14,898	CPAP adherent (≥4 h/night) vs. CPAP non adherent (<4 h/night)	CPAP non-adherent patients showed an increased mortality in comparison with CPAP adherent patients.
Gerves-Pinquie et al., 2022 [82]	Observational study	Adults with OSA starting CPAP	6.6 years	5138	CPAP adherent (≥4 h/night) vs. non-adherent (<4 h/night)	CPAP adherent patients is associated with higher risk of MACEs (death, MI, stroke, HF exacerbation, or revascularization) than the non-adherent patients. The association was stronger in male patients, and those belonging to the excessively sleepy symptom subtype.
Jean-Louis Pépin et al., 2022 [83]	Observational study	Adults patients who had not previously used CPAP and had initiated CPAP.	4 years	176,000	Continued CPAP (over 1 year) vs. CPAP termination (<1 year)	Continuation of CPAP therapy was associated with a significantly lower risk of all-cause death compared with early CPAP therapy termination. Heart failure also was less common in patients who continued CPAP therapy over 1 year.
Mazzotti et al., 2024 [77]	Observational study	Patients with OSA included from 11 USA states (Medicare)	3.1 years	888,835	CPAP vs. usual care	CPAP treatment was associated with lower all-cause mortality and MACE incidence risk (composite of first occurrence of MI, HF, stroke, or coronary revascularization).
Barbé et al., 2012 [84]	Multicenter RCT	AHI ≥ 20, and had no daytime hypersomnolence (ESS < 10).	4 years	725	CPAP vs. usual care alone	In patients with OSA without daytime sleepiness, CPAP treatment compared with usual care did not result in a significant reduction in the incidence of hypertension or CV events (nonfatal MI, stroke, TIA, hospitalization for UA or arrhythmia, HF, or CV death).
McEvoy et al., 2016 (SAVE) [85]	Multicenter RCT	Patients 45–75 y with coronary artery disease or cerebrovascular disease and moderate–severe OSA	3.7 years	2717	CPAP vs. usual care alone	Therapy with CPAP plus usual care, as compared with usual care alone, did not prevent CV events (CV death, MI, stroke, TIA, HF hospitalization for UA). CPAP significantly reduced snoring and daytime sleepiness and improved health-related quality of life and mood.
Peker et al., 2016 (RICCADSA) [86]	Single center RCT	Newly revascularized CAD and OSA (AHI ≥ 15/h) without daytime sleepiness (ESS 10).	4.7 years	244	CPAP (>3 h per night) vs. no-CPAP	Routine prescription of CPAP to patients with CAD with nonsleepy OSA did not significantly reduce long-term adverse cardiovascular outcomes (repeat revascularization, MI, stroke, CV mortality). There was a significant reduction in patients using CPAP ≥ 4 h/night.
De La Torre et al., 2020 (ISAACC) [87]	Multicenter RCT	ACS adult patients with untreated moderate to severe OSAS (mostly non-sleepy), (AHI ≥ 15)	3.35 years	1255	CPAP vs. usual care alone	Among non-sleepy patients with ACS, the presence of OSA was not associated with an increased prevalence of CV events (CV death, MI, stroke, rehospitalization) and treatment with CPAP did not significantly reduce this prevalence.

### 5.2. Randomized Controlled Trials About CPAP Treatment

**Figure 4:**
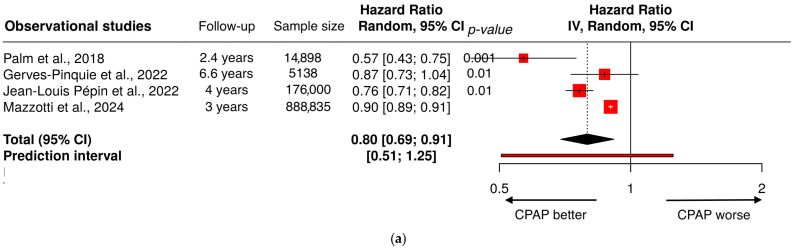

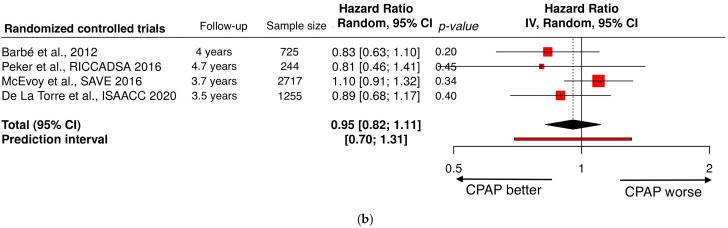
Forest plot showing the risk of major cardiovascular outcomes reported in (**a**) observational studies [77,81,82,83]; (**b**) randomized controlled trials (RCT) [84,85,86,87], comparing patients with OSA treated with CPAP versus those untreated with CPAP. Abbreviations: CPAP, continuous positive airway pressure; CI, confidence interval.

Despite these favorable results in observational studies, large randomized controlled trials (RCTs) have yielded mixed results regarding the benefit of CPAP in reducing major adverse cardiovascular events (MACE), Figure 4.

In a multicenter RCT across 14 Spanish hospitals, Barbé et al. randomized 725 nonsleepy OSA patients (AHI ≥ 20, ESS ≤ 10) to CPAP or usual care, with a median follow-up of 4 years [84]. CPAP did not significantly reduce new-onset hypertension or cardiovascular events. However, adherent users (≥4 h/night) showed a modest risk reduction, suggesting benefit may depend on compliance [84]. In the SAVE trial (2016), 2717 patients aged 45–75 years with moderate-to-severe OSA and established cardiovascular disease, treatment with CPAP improved symptoms and quality of life but did not reduce major cardiovascular events over 3.7 years [85]. A subgroup analysis with adherent users (≥4 h/night), suggested a reduced stroke risk, but no consistent cardiovascular benefit overall. Low adherence (mean 3.3 h/night) may explain the neutral findings, with adherent users showing a signal toward reduced stroke risk [85]. The RICCADSA trial (2016) included 244 revascularized CAD patients with nonsleepy OSA (AHI ≥ 15, Epworth score < 10), showed CPAP did not significantly reduce the composite endpoint of repeat revascularization, MI, stroke, or cardiovascular death, in a follow-up of 57 months [86]. However, patients with good adherence (CPAP ≥ 4 h/night) had a significantly lower risk of events (HR 0.29, 95% CI 0.10–0.86) [86]. Likewise, the ISAACC trial (2020) included 1255 patients hospitalized for ACS with nonsleepy moderate-to-severe OSA (AHI ≥ 15, ESS ≤ 10) [87]. Over a median follow-up of 3.35 years, CPAP did not significantly reduce the primary composite outcome of cardiovascular death, MI, stroke, hospitalization for heart failure, or recurrent ACS/TIA; and OSA itself was not associated with excess risk in this cohort, though CPAP modestly improved sleepiness and blood pressure [87]. Kohler et al. (2011) further demonstrated that CPAP withdrawal leads to rapid recurrence of OSA, sympathetic activation, and hemodynamic stress, though without short-term effects on systemic inflammation or metabolic markers [88].

A consistent limitation across these trials is poor adherence, with average CPAP use often below 4 h/night, likely insufficient to achieve sustained cardiovascular benefit. Subgroup analyses suggest that patients with good adherence may experience reduced risk, underscoring the importance of long-term compliance.

Furthermore, Azarbarzin et al. in a recent pooled analysis of the three major CPAP randomized trials (SAVE, ISAACC, RICCADSA) in 3549 patients with cardiovascular disease and OSA confirmed that overall CPAP did not reduce major adverse cardiovascular and cerebrovascular events (MACCE) [89]. However, when stratified by phenotype, patients with high-risk OSA, defined by greater heart rate responses to apnea/hypopnea or high hypoxic burden, experienced a significant cardiovascular benefit with CPAP, particularly if they were non-sleepy or normotensive, with relative risk reductions up to 35%. In contrast, CPAP was associated with harm in low-risk OSA, likely offsetting benefits in high-risk subgroups and explaining the neutral findings of prior trials. These results suggest that CPAP should be considered a precision therapy for secondary cardiovascular prevention in carefully selected high-risk OSA phenotypes [89].

Strategies such as behavioral interventions, telemonitoring, and individualized mask interfaces may enhance adherence. For CPAP-intolerant patients, alternative modalities—including mandibular advancement devices, positional therapy, hypoglossal nerve stimulation, and investigational pharmacologic agents—are being explored, though their effects on coronary outcomes remain uncertain [90]. Lifestyle interventions are also critical. Weight reduction increased physical activity, and dietary measures improve OSA severity and cardiometabolic health, while bariatric surgery yields profound benefits in apnea burden and metabolic outcomes, though data on long-term coronary risk are limited. Currently, no pharmacological therapy is established, but agents targeting upper airway patency, inflammation, or sympathetic activation are under active investigation [91].

## 6. Conclusions

Obstructive sleep apnea syndrome (OSA) is a common yet underdiagnosed condition with profound implications for coronary artery disease (CAD). Epidemiological evidence consistently demonstrates that OSA independently increases the risk of coronary atherosclerosis, myocardial infarction, and adverse outcomes after revascularization. Several studies highlight intermittent hypoxia, sympathetic activation, oxidative stress, endothelial dysfunction, systemic inflammation, and a prothrombotic state as converging pathways that accelerate coronary pathology.

Although large, randomized trials have not conclusively shown reductions in major cardiovascular events with continuous positive airway pressure (CPAP), these results are tempered by poor treatment adherence. Observational studies and analyses of adherent subgroups suggest that effective therapy can mitigate cardiovascular risk. Lifestyle modification, particularly weight loss, represents a critical adjunctive strategy and addresses both OSA severity and conventional coronary risk factors.

Taken together, OSA should be regarded as a modifiable cardiovascular comorbidity. Systematic screening in patients with CAD, coupled with individualized management strategies that optimize CPAP adherence and address lifestyle risk factors, offers an important opportunity to improve outcomes. Future research should move toward refined phenotyping, novel therapeutic approaches, and integrated cardiovascular–sleep medicine care pathways. Recognizing and managing OSA in the context of CAD is essential for a comprehensive approach to cardiovascular prevention and treatment.

In conclusion, there are several biological pathways linking OSA and increased cardiac arrhythmogenesis propensity. However, the independent association remains based largely on observational studies, and its direction still requires clarification due to the lack of large clinical trials.

## Data Availability

No new data were created or analyzed in this study.

## Supplementary Materials

The following are available online at https://www.mdpi.com/article/10.3390/biomedicines14030515/s1, Figure S1: showing flowchart of study identification and selection for the review on OSA and coronary artery disease (2000–2025). Table S1: showing evidence summary of studies assessing coronary outcomes in OSA vs. non-OSA populations, including OSA definitions, follow-up, endpoints, and reported OR (95% CI); Table S2: showing clinical evidence on CPAP therapy versus no treatment in OSA patients and associated cardiovascular outcomes.
