# Supplementary figures and images for: Obstructive Sleep Apnea and Coronary Artery Disease: An Overlooked Cardiovascular Risk Factor
Source: Biomedicines. 2026 Feb 26;14(3):515. doi: 10.3390/biomedicines14030515 (PMC13023999; doi:10.3390/biomedicines14030515)

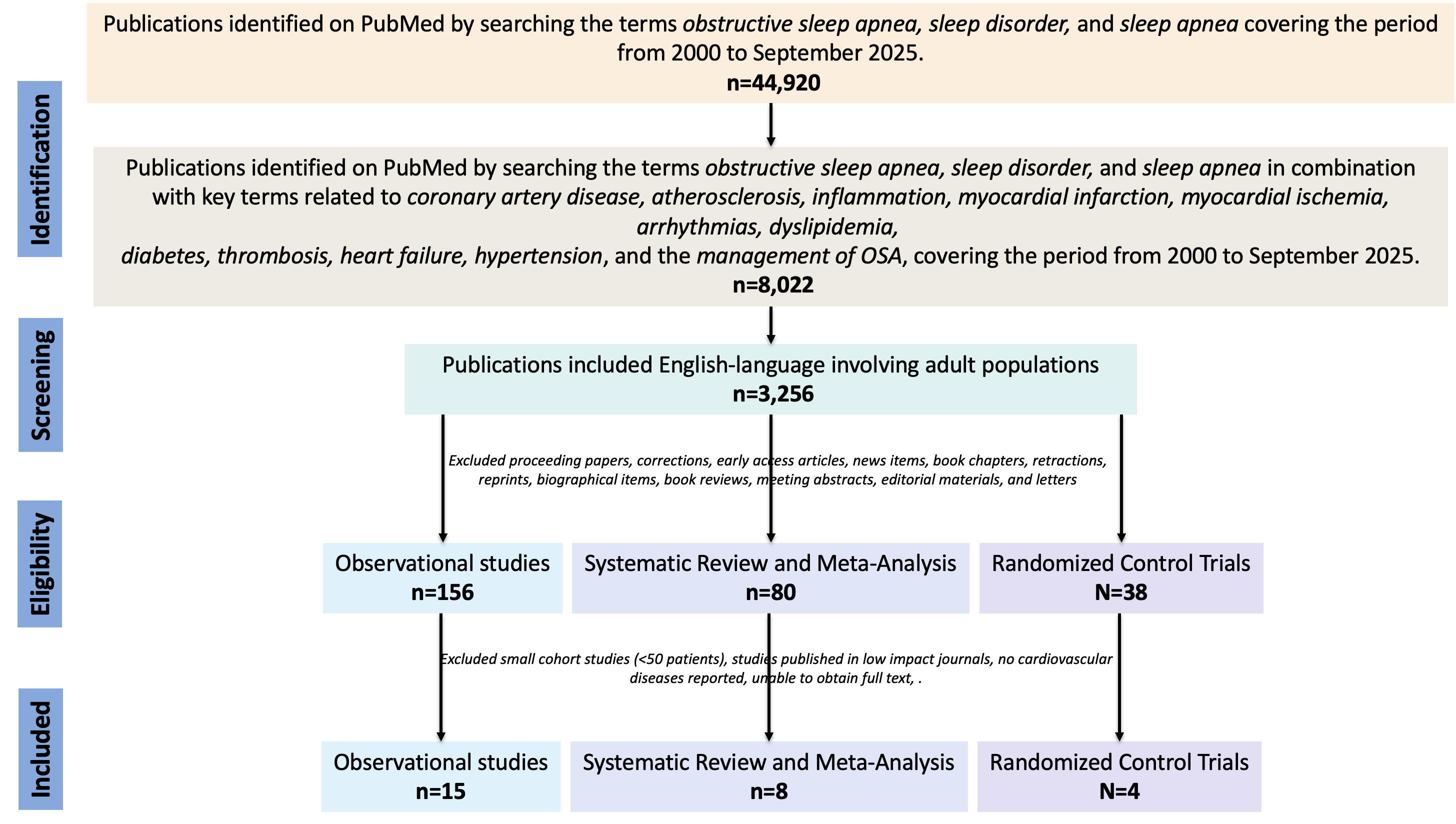

Supplement: Supplementary file 1 [file biomedicines-14-00515-s001.zip › Supplementar material/Figure S1.png]
